# Edited *eukaryotic translation initiation factors* confer resistance against maize lethal necrosis

**DOI:** 10.1111/pbi.14472

**Published:** 2024-10-15

**Authors:** Zhengyu Wen, Fengzhong Lu, Mark Jung, Sabrina Humbert, Lisa Marshall, Craig Hastings, Emily Wu, Todd Jones, Mario Pacheco, Ivan Martinez, L.M. Suresh, Yoseph Beyene, Prasanna Boddupalli, Kevin Pixley, Kanwarpal S. Dhugga

**Affiliations:** ^1^ International Maize and Wheat Improvement Center Texcoco Mexico; ^2^ Current address: KeyGene Inc. Rockville Maryland USA; ^3^ Current address: Maize Research Institute, Sichuan Agricultural University Chengdu China; ^4^ Corteva Agriscience Johnston Iowa USA; ^5^ International Maize and Wheat Improvement Center Nairobi Kenya

**Keywords:** maize lethal necrosis or MLN, virus resistance, eIF4E, gene editing, food security, sub‐Saharan Africa

## Abstract

Maize lethal necrosis (MLN), which is caused by maize chlorotic mottle virus along with a potyvirus, has threatened the food security of smallholders in sub‐Saharan Africa. Mutations in eukaryotic translation initiation factors (eIFs), which also facilitate virus genome translation, are known to confer variable resistance against viruses. Following phylogenetic analysis, we selected two eIF4E proteins from maize as the most likely candidates to facilitate MLN infection. A knockout (KO) of each of the corresponding genes in elite but MLN‐susceptible maize lines conferred only partial protection. Our inability to knockout both the genes together suggested that at least one was required for survival. When we edited (ED) the *eIF4E* genes in Mini Maize, however, the plants with the *eif4e1*‐*KO* became highly resistant, whereas those with the *eif4e2*‐*KO* remained susceptible. Neither of the causal viruses could be detected in the MLN‐inoculated *eif4e1*‐*KO* plants. The *eIF4E2* cDNA in Mini Maize lacked the entire 4th exon, causing a 22‐amino acid in‐frame deletion, which shortened the protein to 198 amino acids. When we introduced mutations in the 4th exon of the *eIF4E2* gene in two elite, MLN‐susceptible lines pre‐edited for an *eif4e1*‐*KO*, we obtained as strong resistance against MLN as in *eif4e1*‐*KO* Mini Maize. The MLN‐inoculated lines with *eif4e1‐KO/eIF4E2‐exon‐4ED* performed as well as the uninoculated wild‐type lines. We demonstrate that the C‐terminal 38 amino acids of eIF4E2 are dispensable for normal plant growth but are required for the multiplication of MLN viruses. Our discovery has wide applications across plant species for developing virus‐resistant varieties.

## Introduction

Diseases and environmental stresses suppress crop yields below their potential levels. Narrowing this gap has been, and will continue to be, the target of plant scientists to ensure food security, particularly in food‐insecure regions (Duvick, 2005; Wulff and Dhugga, 2018).

Maize lethal necrosis (MLN), a viral disease, appeared in Bomet County in Kenya in 2011 and quickly spread to the surrounding countries in Eastern Africa, severely impacting maize production (Boddupalli *et al*., 2020). Most of the existing commercial germplasm at the time was susceptible. On average, MLN reduces grain yield by a quarter, although complete loss in some fields is common (De Groote *et al*., 2016). Sub‐Saharan Africa (SSA), where maize is a staple crop, already has the lowest grain yield in the world (Erenstein *et al*., 2022). MLN has thus further exacerbated food insecurity in this region.

Maize chlorotic mottle virus (MCMV), along with any of the potyviruses, commonly sugarcane mosaic virus (SCMV), causes MLN (Boddupalli *et al*., 2020). Although several QTL are known for SCMV resistance, they are not effective against MLN, as none of these QTLs eliminates SCMV from the plant. If SCMV is present, infection by MCMV would lead to the development of MLN. Resistance against MCMV, as well as against SCMV, is thus required for MLN resistance (Carino *et al*., 2020).

Immediately after its outbreak, CIMMYT established a dedicated MLN Screening Facility in Naivasha, Kenya, in collaboration with the Kenyan Agricultural and Livestock Research Organization to screen the germplasm for resistance to MLN under artificial inoculation (https://mln.cimmyt.org).

An exotic maize line, KS23‐6, developed at the Kasetsart University in Thailand, had earlier been reported to be resistant to MLN (Jampatong *et al*., 2010; Jones *et al*., 2018). A single QTL on chromosome 6 (C6QTL) was found to have a major effect on MLN resistance (Boddupalli *et al*., 2020; Jones *et al*., 2018; Murithi *et al*., 2021). The C6QTL was introgressed into an array of elite, MLN‐susceptible parental lines of commercial hybrids by forward breeding (Awata *et al*., 2021). Field screening in Naivasha was key in developing the MLN‐tolerant hybrids.

Indispensable as forward breeding is for continued, incremental crop improvement, it is a time‐ and resource‐intensive process. In cases involving introgression of a simple trait from an exotic relative, the most common source of the desirable gene variants, undesirable genes from the donor parent continue to persist in the converted lines (Dhugga, 2022; Wulff and Dhugga, 2018). Alternative technologies, in contrast, could help expedite the process of gene transfer for the simply inherited traits (Wulff and Dhugga, 2018). Gene editing, for example in combination with genotype‐independent plant transformation technology, can be effective in the precise introduction of targeted changes directly into key commercial lines, eliminating the need for backcrossing (Dhugga, 2022; Lowe *et al*., 2016, 2018a; Svitashev *et al*., 2015). This technology is suitable when the trait of interest is simply inherited, gene‐trait relationship is established, the gene sequence is known and preferably a simple knockout results in the desirable phenotype (Dhugga, 2022).

To avoid the host's defence, viruses are known to form invaginations, also referred to as spherules, in various cell membranes where they assemble replicase complexes and utilise their host's protein translation machinery to make their own proteins (Li *et al*., 2023; Nagy, 2016; Sanfacon, 2015; Wolff *et al*., 2020). MCMV forms spherules in the peroxisomal membrane, whereas SCMV does so in the endoplasmic reticulum (Laliberté and Sanfaçon, 2010). To translate their own respective genomes, viruses have evolved non‐canonical mechanisms to recruit the protein translation machinery of their hosts (Simon and Miller, 2013; Sorokin *et al*., 2021). In eukaryotes, a 5′‐cap consisting of 7‐methyl guanosine (m7G) attached through a 5′‐5′ linkage to the mRNA acts as a recognition signal for the translation initiation complex. A viral genome‐linked protein (VPg) in potyviruses competes with the m7G‐capped host mRNA, whereas cap‐independent translation enhancers (CITEs) in the 3′ untranslated region (UTR) of the MCMV RNA, which lacks the VPg protein, are known to assist in its docking to the initiation complex (Simon and Miller, 2013). A specific CITE smaller than 200 nucleotides in the 3’ UTR of MCMV has recently been reported to interact with the eIF4E protein (Carino *et al*., 2020).

In the absence of known genes for MLN resistance, we decided to extend the knowledge from other plant species where specific genes had been shown to be related to virus susceptibility. Mutations in the host factors that are constituents of the eukaryotic translation initiation/elongation (eIF/eEF, referred to hereon as eIF for simplicity) complex were known to enable virus resistance in other plant species but not maize (Atarashi *et al*., 2020; Piron *et al*., 2010; Ruffel *et al*., 2006; Sanfacon, 2015). The eIF proteins in each subfamily are generally encoded by multiple genes, making it difficult to completely block virus replication (Bastet *et al*., 2017). The virus may, however, have a stronger affinity for one or the other eIF4E paralog for the translation of its genome (Wang *et al*., 2013).

We compared the known eIF proteins from other species where their mutant versions conferred virus resistance to identify the corresponding maize proteins. Based on phylogenetic analysis, we narrowed down the maize eIF factors to where the knockouts were most likely to confer resistance against MLN. We also discovered a novel genetic variant for one of the eIF genes, which, when combined with a knockout of the other member, caused strong MLN resistance in an experimental maize line. A knockout of one eIF combined with a modified second factor in commercial lines resulted in complete resistance against MLN. These results are reported in this communication. Our discovery has opened a new path to introduce virus resistance directly in elite germplasm in a relatively short period of time and should be applicable across other plant species.

## Results and discussion

### Identification of maize eukaryotic translation initiation/elongation factors

In the continuing host‐pathogen battle, one of the means the host acquires resistance is through limiting the translation of the viral genome. Mutations in the eukaryotic translation initiation/elongation factors (eIF), which are components of the translation initiation/elongation complex, are known to confer variable levels of resistance against viruses (Hoffie *et al*., 2021; Sanfacon, 2015; Simon and Miller, 2013). No reports were available in maize, however, particularly for MLN.

To identify maize paralogs of the known eIF proteins for virus resistance, we performed phylogenetic analysis of the maize eIF proteins along with those from other plant species (Figure S1). This approach proved useful previously in assigning specific function to genes within a family (Appenzeller *et al*., 2004; Dhugga, 2001). Four of the 17 maize proteins, two each from the eIF4E and eIF(iso)4E subfamilies, grouped in a clade with the highest frequency of eIF proteins from non‐maize plant species where their mutants were known to confer virus resistance (Figure S1).

### Editing the genes for the eIF4E and eIF(iso)4E factors

**Figure 1:**
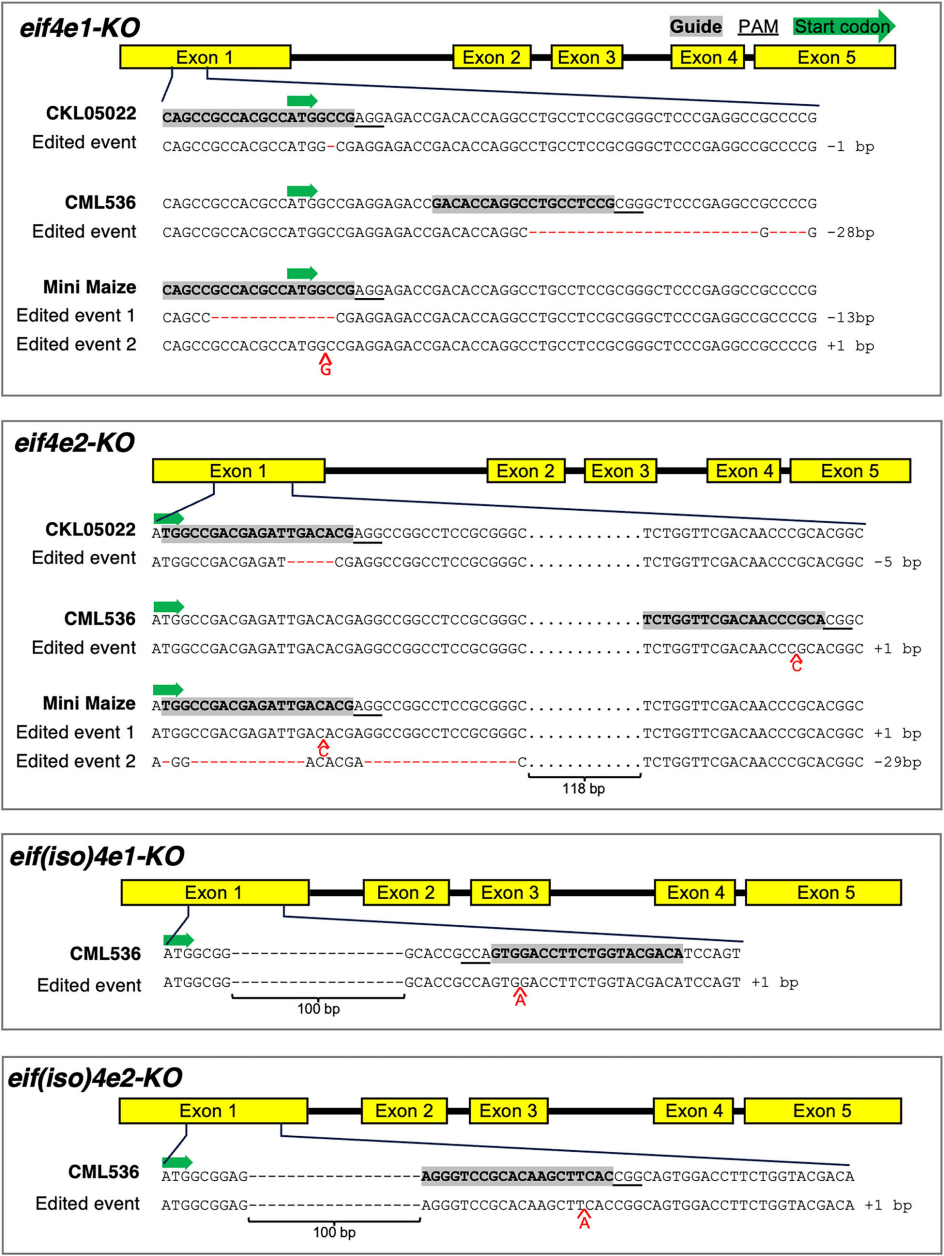
Editing of *eIF4E* and *eIF(iso)4E* genes in elite maize lines CKL05022 and CML536, and Mini Maize. The guide RNAs were targeted to the first exon. Nucleotide deletions are depicted with red dashes and additions with respective bases under each sequence. The listed events were screened for response to MLN after artificial inoculation. For each genotype, all the selected events were apparent knockouts from frameshift mutations.

**Figure 2:**
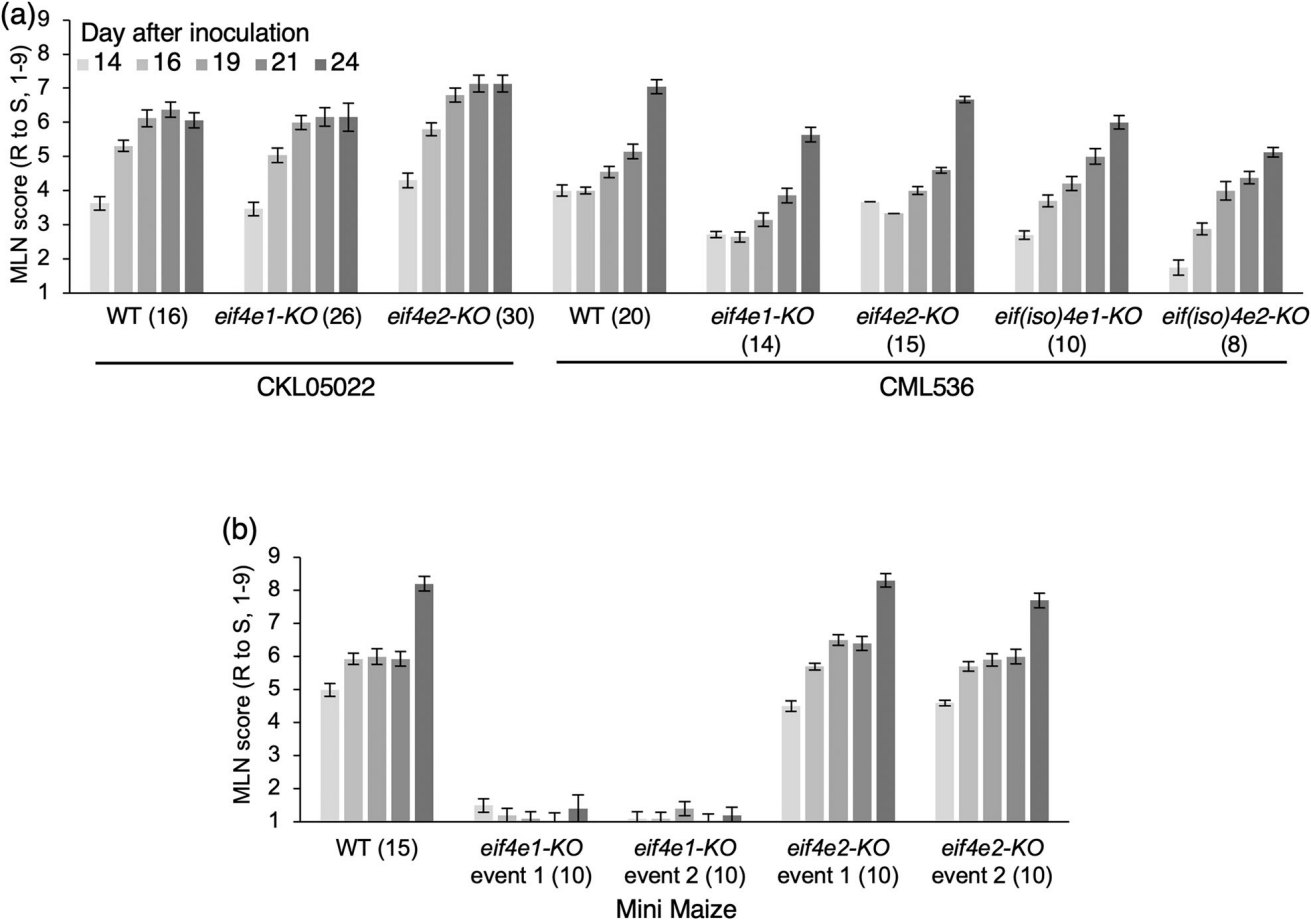
Screening of parental lines and gene‐edited variants for MLN resistance of elite CIMMYT lines (a) and Mini Maize (b). MLN symptoms were recorded starting 14 days after MLN inoculation on a scale of 1 (no symptoms, resistant or R) to 9 (plant death, susceptible or S). Error bars represent standard error estimates, and the numbers in parentheses are the biological repeats (plants). Molecular details of gene editing for each event are shown in Figure 1. The method for scoring MLN is described in https://mln.cimmyt.org. The least score for MLN screening is set to 1, which corresponds to no disease symptoms. The SE estimates for *eif4e1‐KO* in Mini Maize result from minor damage to the leaves, which can be physical in nature but is recorded as a score higher than 1.

**Figure 3:**
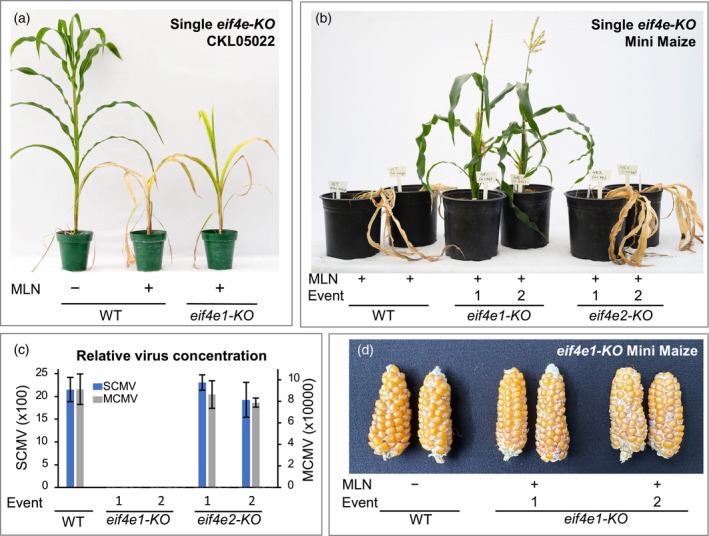
Effect of knocking out *eif4e* genes on resistance against maize lethal necrosis (MLN) in an elite maize line and Mini Maize. (a) Response of CKL05022 knocked out (*KO*) for *eif4e1* to MLN 45 days after inoculation (dai). (b) Response of Mini Maize with *eif4e1‐KO* or *eif4e2‐KO* to MLN (40 dai). (c) SCMV and MCMV quantification by ELISA in MLN‐inoculated Mini Maize plants. (d) Ears of self‐pollinated Mini Maize.

We targeted a single guide RNA (sgRNA) to each of the two genes in the *eIF4E* subfamily in two elite MLN‐susceptible CIMMYT maize inbred lines, CKL05022 and CML536 (Figures 1 and S2) (Masuka *et al*., 2017). Further, we edited the *eIF(iso)4E1* and *eIF(iso)4E2* genes in CML536 (Figures 1 and S3). Edited lines homozygous for respective mutant alleles were screened in the greenhouse for response to artificial MLN inoculation (Gowda *et al*., 2015). Single knockouts (KO) of each of the genes conferred partial resistance in both the lines (Figure 2a). Although the edited plants produced several additional leaves as compared to the wildtype after MLN inoculation, none of the plants survived to flowering stage (Figure 3a). We were unable to recover double knockouts for the *eif4e1*/*eif4e2* combination despite multiple attempts with either co‐transformation using the guides for both the genes or using the *eIF4E2* guide to transform the *eif4e1‐KO* events and vice versa. This suggested that at least one or the other paralog from the *eIF4E* subfamily was required for plant survival.

The virus may prefer one or the other paralog (or member) of the eIF4E or eIF(iso)4E subfamilies, so knocking out various members may provide different levels of resistance (Wang *et al*., 2013). However, as long as one of the family members, which is required for plant survival, is functional, the virus can still replicate. This would result in partial resistance at best.

### Editing *eIF4E* genes in Fast Flowering Mini Maize delivers complete MLN resistance

Fast Flowering Mini Maize (referred to as Mini Maize hereon) is a dwarf experimental line with a short lifecycle, making for a relatively quick testing of the effect of gene alteration on phenotype (McCaw *et al*., 2016). When we knocked out the *eIF4E1* gene in this line, the edited plants were highly resistant to MLN (Figures 2b and 3b). In agreement, we did not detect either MCMV or SCMV in the leaf tissue of the MLN‐inoculated plants (Figure 3c). The plants with the edited *eif4e1* gene went on to produce normal‐looking ears like the wild‐type plants (Figure 3d). In contrast, the *eif4e2‐KO* plants exhibited only marginal improvement in resistance (Figure 3b). This suggested that the eIF4E2 protein was altered in Mini Maize, which allowed for normal plant growth but no longer facilitated the translation of the viral genome in the absence of eIF4E1.

**Figure 4:**
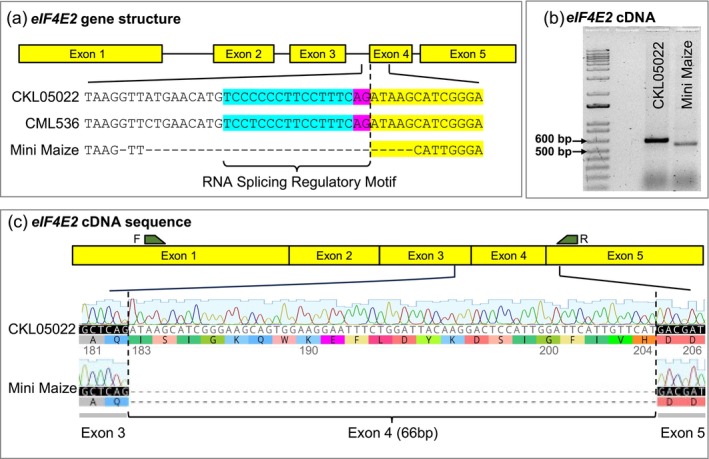
Sequence comparison of Mini Maize *eIF4E2* against CML536 and CKL05022. (a) Comparison of genomic sequence of *eIF4E2* among Mini Maize, CKL05022 and CML536. The deletion across the intron/exon junction in Mini Maize is marked by dashes. The regulatory motif for RNA splicing between the 3rd intron and 4th exon is highlighted in blue and purple. (b) RT‐PCR of *eIF4E2*. (c) Sanger sequences of *eIF4E2* cDNA with the missing nucleotides in Mini Maize marked by dashes.

A comparison of the genomic sequences of *eIF4E2* revealed a novel deletion encompassing the 3′ region of the 3rd intron and the 5′ region of the 4th exon in Mini Maize as compared to CKL05022 and CML536 (Figure 4a). The deletion included the pyrimidine‐rich motif that preceded the splicing motif, AG, at the intron‐3/exon‐4 junction along with five nucleotides from the 4th exon (Lim and Burge, 2001). The cDNA for the *eIF4E2* gene from Mini Maize was distinctly smaller than that from CKL05022 (Figure 4b). Sanger sequencing revealed that the entire exon 4 was spliced out from the Mini Maize *eIF4E2* cDNA (Figure 4c). The destruction of the intron/exon junction motif apparently caused alternative RNA processing where the remainder of exon‐4 was spliced out as part of the larger intron that encompassed the 3rd and 4th introns. The excision of the 4th exon shortened the predicted eIF4E2 protein by 22 amino acids but without affecting its isoelectric point, which might be important in protein‐protein interactions (size of the truncated protein ~22 kDa, *pI* 5.8). The native protein is 220 amino acids long (24.6 kDa, *pI* 5.8 for CML536).

With the goal of utilising this naturally occurring variation to introduce MLN resistance directly into elite lines by knocking out just the *eIF4E1* gene, we screened more than 40 genetically diverse CIMMYT maize lines, including those developed in Africa. None lacked the 4th exon.

### The in‐frame deletion in *eIF4E2* conditions MLN resistance

**Figure 5:**
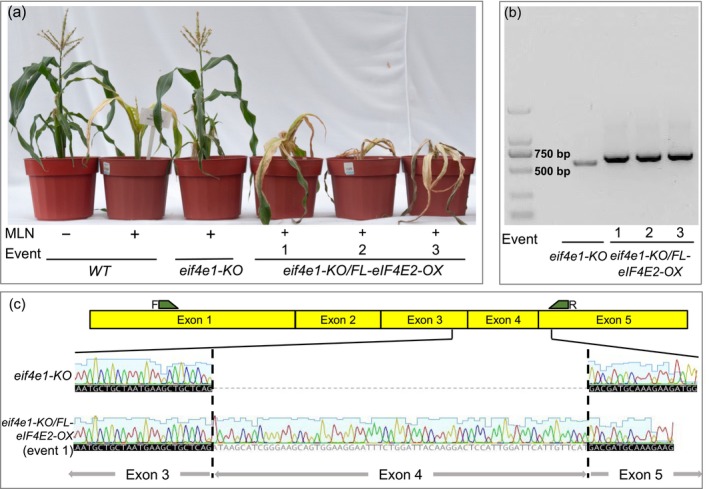
Effect of overexpression of full‐length (FL) *eIF4E2* cDNA from CML536 in Mini Maize knocked‐out for *eif4e1* on susceptibility to MLN. (a) Response to MLN 40 days after inoculation of wild‐type, *eif4e1‐KO* and FL‐*eIF4E2* cDNA overexpressing (OX) events. (b) RT‐PCR of young leaf tissue from wild‐type Mini Maize with *eif4e1‐KO* and three *eIF4E2*(OX) events. The absence of the native, truncated cDNA in the overexpressing events suggests disproportionate expression of the full‐length cDNA under the control of an ectopic promoter. (c) Comparison of Sanger sequence of *eIF4E2* cDNA from wild‐type Mini Maize with *eif4e1‐KO* and an event overexpressing the full‐length cDNA from CML536.

To determine whether the modified *eIF4E2* gene in Mini Maize was indeed responsible for MLN resistance, we overexpressed the full‐length *eIF4E2* cDNA corresponding to the inbred line CML536 in Mini Maize pre‐edited for *eif4e1‐KO* (Figure 5). We confirmed the expression of the transgenic cDNA by RT‐PCR in multiple events and also by Sanger sequencing (Figure 5b,c). The plants overexpressing the *eIF4E2* cDNA were highly susceptible to MLN, which proved that the in‐frame deletion in Mini Maize *eIF4E2* was indeed responsible for MLN resistance (Figure 5a).

The truncated eIF4E2 protein was apparently sufficient for normal plant growth in the absence of eIF4E1, but it did not facilitate the interaction of the MCMV or SCMV genome with the eIF complex. We threaded the structures of the full‐length and truncated versions of the maize eIF4E2 proteins based on the structure from the human eIF4E (Coutinho de Oliveira *et al*., 2019; Peter *et al*., 2015; Volpon *et al*., 2006). The binding of the viral genome‐linked protein (VPg) from potato virus Y involves the C‐terminal domain of the eIF4E protein, which does not overlap with the binding site of eIF4G, another protein that is involved in forming the translation initiation complex (Figures S4 and S5). The truncation or elimination of the C‐terminal folds in the maize eIF4E2 protein makes it unrecognizable to both SCMV, a potyvirus, and MCMV, a Tombusvirus.

Unlike SCMV, MCMV does not bind the eIF4E protein through a VPg but does so directly via the 3’‐CITEs, which form various types of secondary structures (Carino *et al*., 2020; Simon and Miller, 2013). Viral VPg‐bound RNAs are known to compete with the 5′‐m7G‐capped eukaryotic mRNAs for binding to the eIF4E protein (Simon and Miller, 2013). The maize mRNAs, however, must still be able to bind the truncated eIF4E. The VPg from SCMV has a molecular mass of approximately 21 kDa (accession: AZM65781.1) as compared to 0.3 kDa of m7G, so would require a larger surface to form a stable complex with the eIF4E protein (Figure S5). Based on the crystal structure of the murine eIF4E bound to 7‐methyl‐GDP, a number of amino acid residues were identified that were conserved across diverse species, including wheat (Marcotrigiano *et al*., 1997). None of these conserved residues is found in the C‐terminal domain that is partially or completely deleted in Mini Maize or edited variants in the elite lines (Figure S4b). In contrast, two of the conserved residues, a histidine and a lysine, required for VPg binding to the eIF4E protein happen to be in this domain (Figure S4a). This likely explains why the truncated eIF4E is still able to recognise the maize mRNA but not the viral RNA. It is quite possible that the C‐terminal domain interacts with the genomes of other viruses as well. That would make it a suitable target to exploit for introducing broad‐spectrum resistance. Our discovery should bring other plant species, even the ones that contain only a single copy of the *eIF4E* gene, within the scope of gene editing to confer virus resistance.

### Edited *eIF4E2* gene in combination with a knocked out *eif4e1* gene confers strong MLN resistance in elite CIMMYT maize lines

**Figure 6:**
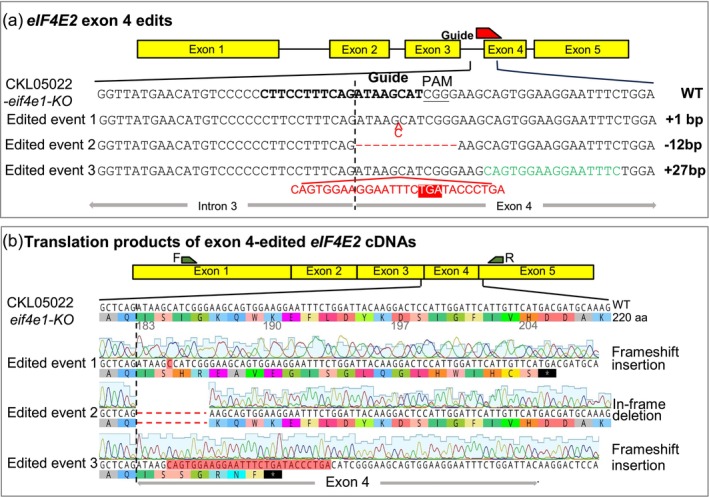
Sequences of the edited alleles of *eIF4E2* in CKL05022 pre‐edited for *eif4e1‐KO.* (a) Sequences of the edited exon 4 from *eif4e1‐KO/eIF4E2‐exon‐4ED* double edits. A vertical dotted line is drawn at the intron/exon junction. (b) Sanger sequencing of the cDNA of *eIF4E2‐exon‐4* edited events from (a). These double‐edited events were generated by transforming the *eIF4E2‐exon‐4* guide RNA into the construct‐free CKL05022 *eif4e1‐KO* event from Figure 1. An insertion caused a shift in the open reading frame starting with amino acid 185 (event 1), an in‐frame deletion (marked with red dashes) shortened the ORF by four amino acids (event 2) and an insertion of 27 nucleotides, 18 of which were identical to the gene itself starting at 10 nucleotides downstream of the insertion site (green font), shifted the frame starting with the amino acid 185 (event 3). In events 1 and 3, the ORF was prematurely terminated because of the stop codons that resulted from the frameshift (asterisks in black background).

Our original goal was to introduce MLN resistance directly into elite CIMMYT maize lines from Africa. Encouraged by the results from Mini Maize, we thus targeted an sgRNA to the 5′ end of the 4th exon of the *eIF4E2* gene in CKL05022 and CML536 where the *eif4e1* gene had already been knocked out (Figure 1). A range of variants resulted, and the expression of edited *eIF4E2* in these events was confirmed by RT‐PCR (Figure S6a). Most of the variants contained frameshift mutations close to the 5′‐end of the 4th exon (Figures 6a and S6b). For CKL05022, for example, the edit in one of the events selected for further study caused a single nucleotide deletion, another a 12‐nucleotide deletion resulting in an in‐frame removal of 4 amino acids from the translated protein, and yet another a 27‐nucleotide insertion that shifted the reading frame as well as introduced a premature stop codon (Figure 6a). For CML536, one of the selected events had a single nucleotide addition, the second a single‐nucleotide deletion and the third one had an eight‐nucleotide deletion that included the G of the AG intron/exon junction (Figure S6b). Disruption of the intron/exon splice junction suggested the next available AG motif in the 4th exon might serve as the new splice junction.

Instead of relying on theoretical prediction, we performed RT‐PCR followed by Sanger sequencing to determine whether the NextGen sequences of the genomic amplicons of the edited events matched the cDNA sequences. For CKL05022, the deletion of 12 nucleotides beginning with the first nucleotide after the AG intron/exon splice junction was preserved in the cDNA (Figure 6b). However, for CML536, where the splice junction was disrupted, instead of the next AG downstream of the disrupted intron‐3/exon‐4 splice junction, it was the second AG where splicing occurred (Figure S6b). The mature transcript was shorter by 18 nucleotides, causing an in‐frame deletion of six amino acids (Figure S6b). Excision of the whole exon‐4 in Mini Maize, where only five nucleotides from exon‐4 were included in the naturally occurring deletion, may be attributed to the deletion of the polypyrimidine‐rich motif that preceded the AG splice site (Lim and Burge, 2001). This motif was intact in the CML536 edit. It is not clear, however, why splicing occurred at the second AG motif and not the first available one (Figure 6Sb).

**Figure 7:**
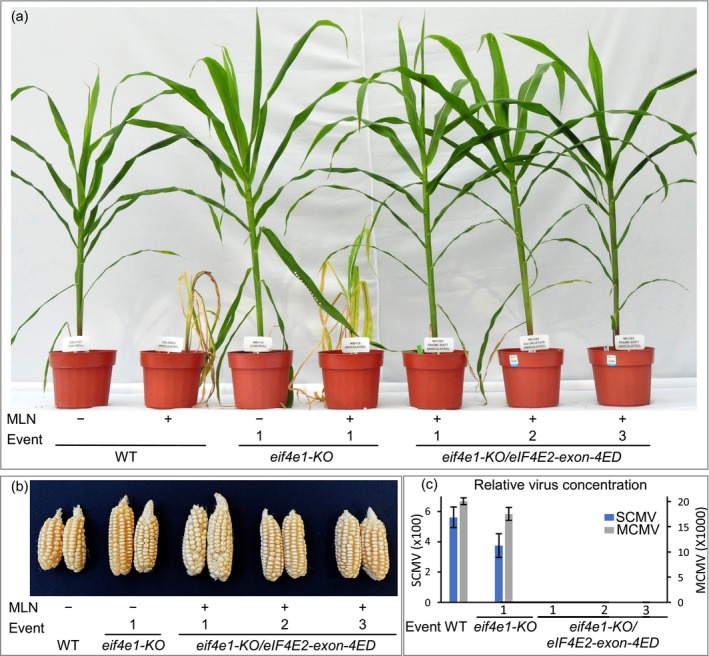
Effect of double edits of *eif4e1‐KO* and *eIF4E2‐exon‐4ED* on MLN resistance in the elite CIMMYT inbred line CKL05022. *ED*, edited. (a) Response of *eif4e1‐KO/eIF4E2‐exon‐4ED* double edits to MLN 45 days after inoculation. (b) Self‐pollinated ears of *eif4e1‐KO/eIF4E2‐exon‐4ED* double edits and control plants. (c) Quantification of SCMV and MCMV 20 days after MLN inoculation. *ED,* edits 3' of the beginning of the 4th exon.

As the double‐edited plants grew normally, in the backdrop of our inability to obtain double knockouts of the *eIF4E* genes, all the variants of *eIF4E2* edited in the 4th exon must be functional proteins that allow normal plant growth. We thus refer to the double edits as *eif4e1‐KO*/*eIF4E2‐exon‐4ED* (Figure 7).

**Figure 8:**
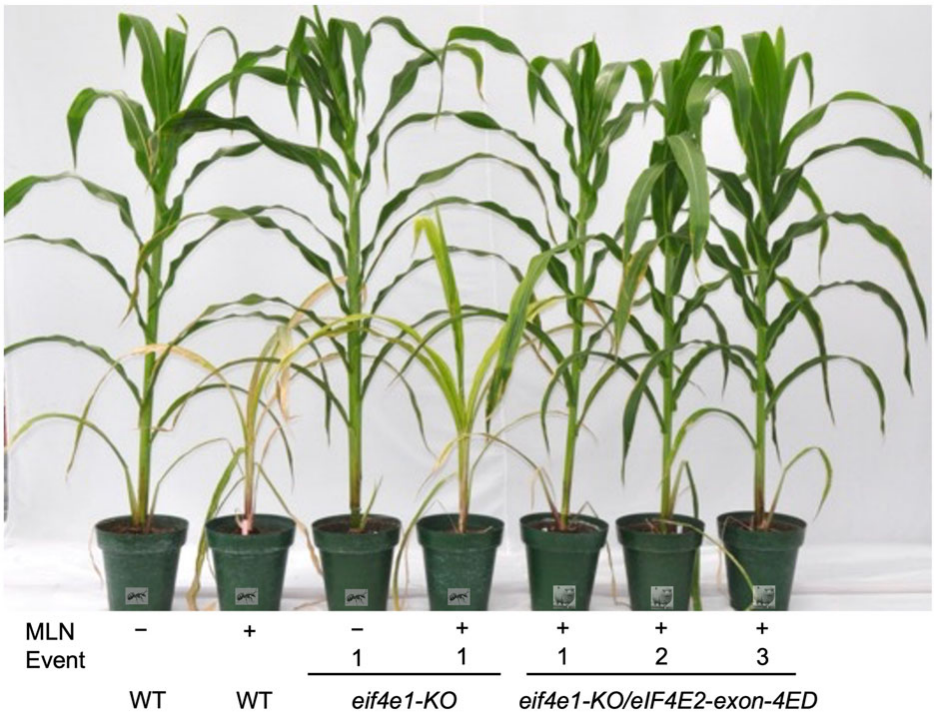
Effect of single (*eif4e‐KO*) or double (*eif4e1‐KO/eIF4E2*‐*exon‐4ED)* gene edits on response to MLN in CML536. The double‐edited events were generated by transforming the *eIF4E2‐exon‐4* guide into a construct‐free CML536‐*eif4e1‐KO* event from Figure 1. Events 1 and 2 in double edits were single‐base indel mutants, shifting the frame starting with the 3rd amino acid of the 4th exon. Event 3 was an in‐frame mutant with the first six amino acids corresponding to the 4th exon deleted (see Figure S6 for details). All three events were equally resistant to MLN.

We screened several double‐edited variants for resistance against MLN in the greenhouse. Whereas the unedited parental lines died at 7–8 leaf stage, frameshift mutations, as previously discussed, in either *eIF4E1* or *eIF4E2* produced several additional leaves before the plants died (Figures 3a, 7a and 8). For reference, the ear is born at the 9th or 10th node aboveground in tropical maize lines, which generally have 17–18 total aboveground leaves. The plants with the *eif4e1‐KO*/*eIF4E2‐exon‐4ED* edits looked normal with no sign of the disease upon MLN inoculation and went on to produce normal‐looking ears that were similar to the uninoculated parental lines (Figure 7a, b). Neither MCMV nor SCMV could be detected in the MLN‐inoculated, double‐edited plants with ELISA assays, whereas leaves of the unedited plants or the ones with only *eif4e1‐KO* contained high levels of these viruses (Figure 7c).

The *eif4e1‐KO*/*eIF4E2‐exon‐4ED* double edits in CML536 were just as resistant to MLN as the double edits of CKL05022 (Figure 8). These results provide independent confirmation that the truncated or modified eIF4E2 protein is not recognised by either of the MLN viruses for the translation of their respective genomes.

**Figure 9:**
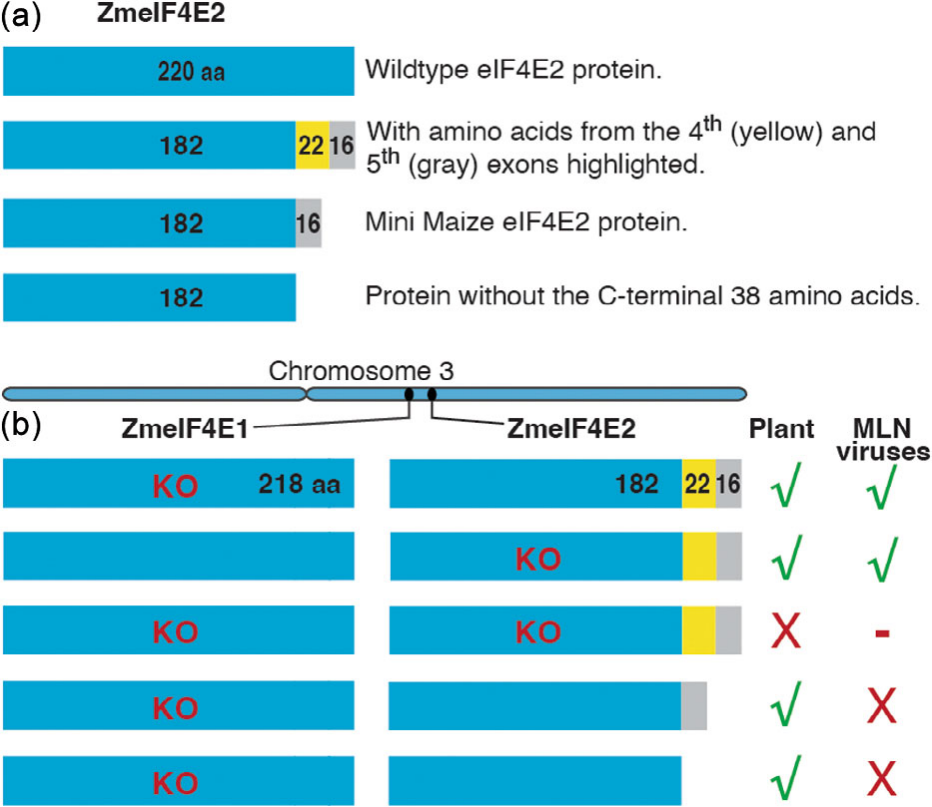
Wild‐type eIF4E2 protein and its natural and edited variants in maize (a), and the effect of different combinations of *eif4e1‐KO* and *eIF4E2ED* variants on viral replication and plant growth. KO, knockout; X, no growth; − no host; √, normal growth. The numbers in the respective horizonal bars are for the amino acids. (a) The wild‐type eIF4E2 in maize is 220 amino acids long. A naturally occurring variant in Mini Maize has an in‐frame deletion of 22 amino acids corresponding to the 4th exon. Some of the edited variants in eIF4E2 from an elite line, CKL05022, are shorter by nearly the entire stretch of C‐terminal 38 amino acids. (b) Effect of combinations of a knocked‐out eIF4E1 (218 amino acids long) and different variants of eIF4E2 on MLN resistance. Aside from the naturally occurring variant of eIF4E2 in Mini Maize, any frameshift mutation in the 4th exon resulted in complete MLN resistance. In addition to frameshift mutants, we obtained at least two in‐frame mutants that lacked either the first four amino acids (181–184) in CKL05022 (Figure 6, event 2) or the first six amino acids (183‐188) in CML536 (Figure S6, event 3) corresponding to the 4th exon that were completely resistant to MLN. The C‐terminal 38 amino acids appear to constitute a domain that is required for recognition by the viruses to translate their proteins but is not necessary for the translation of the maize proteins. The two eIF4E proteins differ only at two positions in the C‐terminal 38‐aa stretch (Figure S4). Designing a single guide RNA around the junction of the 3rd intron/4th exon for both the *eIF4E1* and *eIF4E2* genes would expedite the introduction of MLN resistance in susceptible elite lines. These findings could be extrapolated to other plant species to develop broad‐spectrum resistance against viruses.

In the absence of a functional eIF4E1, the eIF4E2 protein lacking the C‐terminal 38 amino acids blocked the replication of both viruses (Figure 9). Regardless of whether it was a frameshift mutation or an in‐frame deletion of a few amino acids, any alteration in *eIF4E2* starting with the 4th exon in the background of *eif4e1‐KO* sufficed to block virus replication without affecting plant growth. From the translated product of *eIF4E2*, which is 220‐aa long, the C‐terminal 38‐aa are apparently dispensable for normal plant growth (Figure 9).

This C‐terminal stretch of amino acids corresponds to a domain known to interact with the VPg in human eIF4E (Coutinho de Oliveira *et al*., 2019). A tyrosyl group in VPg covalently binds the 5′ end of the potyvirus RNA and competes with the m7G‐capped host mRNA. This may account for SCMV resistance; however, our results show that this domain must also be required for the recognition of 3’‐CITEs from the MCMV genome (Coutinho de Oliveira *et al*., 2019; Simon and Miller, 2013; Sorokin *et al*., 2021).

The C‐terminal 38‐amino acid domain is nearly identical between eIF4E1 and eIF4E2 (Figure S4), suggesting that an alteration in this domain in eIF4E1 may also make it unrecognisable to the viral genome (Figure 9). Instead of knocking out one of the *eIF4E* genes and modifying the other in the 4th exon, both genes could be simultaneously targeted only in the 4th exon to generate double edits for MLN resistance directly in the commercial, MLN‐susceptible lines.

Our study demonstrates that knocking out eIF4E1 combined with the introduction of mutations in the C‐terminal 38‐amino acid stretch of eIF4E2 ensures normal plant growth yet confers strong resistance against MLN (Figures 3, 7 and 8). We have further demonstrated that we can accomplish this directly in elite maize lines that are susceptible to MLN (Figures 7 and 8), thus eliminating the process of backcrossing, which would be required were the initial edits made in an experimental line (Dhugga, 2022). The whole process, from the discovery of genes to testing of the edited elite maize lines for MLN resistance, was accomplished in less than 3 years.

The closely spaced *eIF4E1* and *eIF4E2* genes are located near the centromere on chromosome 3, in a low recombination region (Guan *et al*., 2017). Gene editing made it possible to alter both these genes, which would be challenging if the mutations in each gene were to be stacked genetically. The process of introgressing this edited gene pair into other lines would also be simpler, as it would predominantly segregate as a single locus.

The next step is to test the *eIF4E*‐edited lines in the MLN screening facility in Naivasha, Kenya, under artificial inoculation (Boddupalli *et al*., 2020). Kenya recently published the guidelines on growing gene‐edited plants in open fields (NBA‐Kenya, 2022). Almost all the CIMMYT‐derived drought‐tolerant hybrids commercialised before the outbreak of MLN were susceptible. Now that we have established a procedure to introduce strong resistance against MLN directly in elite lines in a relatively short period of time, a pipeline could be established to edit a wider array of MLN‐susceptible lines and turn them around in 2–3 years for hybrid production. Gene editing presents a unique opportunity to help protect food security and support smallholders in Eastern Africa.

### Experimental procedures

#### Gene sequences and analysis

Maize eukaryotic translation initiation/elongation factor (eIF/eEF) sequences were downloaded from the Maize GDB database (https://www.maizegdb.org). The eIF proteins from other plant species were downloaded based on the information from the published reports (Albar *et al*., 2006; Ashby *et al*., 2011; Chen *et al*., 2021; Gao *et al*., 2004; Hashimoto *et al*., 2016; Hebrard *et al*., 2010; Hofinger *et al*., 2011; Hwang *et al*., 2009, 2013; Lee *et al*., 2010; Li *et al*., 2016; Ling *et al*., 2009; Luan *et al*., 2016; Naderpour *et al*., 2010; Nieto *et al*., 2006; Piron *et al*., 2010; Ruffel *et al*., 2002, 2005; Sanfacon, 2015; Shi *et al*., 2017; Shopan *et al*., 2020; Zeenko *et al*., 2002). Phylogenetic analysis was carried out using Geneious Prime software (Neighbour Joining Method, Global Alignment with Free End Gaps, PAM 160, with gap opening penalty set to 12 and gap extension penalty set to 3).

Genomic sequences for the *eIF4E* and *eIF(iso)4E* as well as those for *eIF4G* and *eIF(iso)4G* were amplified from the genomic DNA of respective genotypes for further studies.

#### Plant material

Elite maize inbred lines CKL05022 and CML536 were obtained from CIMMYT germplasm bank. Fast Flowering Mini Maize (TX40J), which we refer to as Mini Maize for simplicity, was obtained from the Maize Genetics Cooperation Stock Center (McCaw *et al*., 2016). All plants were grown in the greenhouses at CIMMYT in Mexico.

#### Gene editing and overexpression

Gene editing constructs, *Agrobacterium* transformation and NextGen sequencing of gene‐edited lines were as described previously (Lowe *et al*., 2018b; Shi *et al*., 2015; Svitashev *et al*., 2015). Briefly, guide sequence was inserted into a gene editing construct containing a *ZmAxig1pro:ZmWus2* cassette, a *ZmUbi1pro:SpCas9* cassette and a *ZmU6pro:gRNA* cassette and transformed into 2 mm immature maize embryos extracted from the ears approximately two weeks after pollination via the LBA4404(THY‐) strain of *A. tumefaciens* containing the *pVIR9* plasmid. This was followed by selection on G418 to select the transformed somatic embryos. Single‐guide RNA (sgRNA) sequences are listed in Table S1.

We generated about 50 events per transformation and sequenced 20 for the edits. Leaf tissue was sampled from the individually regenerated seedlings, and approximately 200 bp genomic regions covering the sgRNA target site were amplified, barcoded and subjected to Illumina sequencing (Lowe *et al*., 2018b; Shi *et al*., 2015; Svitashev *et al*., 2015). T0 plants were self‐pollinated, and the events homozygous for the edits were advanced. Construct‐free T1 seedlings were advanced to produce T2 seed. Transformation efficiency ranged from 10% for CKL05022 to 80%–90% for CML536 and Mini Maize. Editing efficiency ranged from 90% to 100% and even the occasional wild‐type event may have been an escape from selection in tissue culture. On average, approximately 30% of the edited T0 events were homozygous for the edits. Nearly 40% of the edits were from a single nt deletion, ~55% from 2 nt or longer deletions and rare ones from nt additions or substitutions.

The response of the edited lines to MLN was evaluated in homozygous T2 plants.

To generate double gene edits, for example, *eif4e1‐KO/eIF4E2‐exon‐4* events, *eif4e1‐KO* events from CKL05022 and CML536 lines were selected for further transformation with the construct containing the sgRNA targeted to exon‐4 of the *eIF4E2* gene. For overexpression of the full‐length *eIF4E2* gene, a construct containing a full‐length *eIF4E2* cDNA corresponding to the CML536 gene was synthesised (GenScript) and transformed into the *eif4e1‐KO* Mini Maize under the control of the *ZmUbi1‐intron* promoter.

Another small family, eIF4G, is closest to the known proteins from several plant species where their mutants have been shown to confer virus resistance (Macovei *et al*., 2018; Rubio *et al*., 2019; Rupp *et al*., 2019; Wang *et al*., 2021). We have edited all four of the individual genes for this family in Mini Maize and are currently in the process of screening the mutants for resistance against MLN. These results will be the subject of a separate report.

#### Greenhouse MLN phenotyping

Protocols for virus maintenance, inoculation and scoring of MLN symptoms were adapted from the MLN information portal at CIMMYT (https://mln.cimmyt.org). SCMV isolate, which originated in Irapuato, Guanajuato (Mexico), was obtained from CINVESTAV (Mexico City). MCMV isolate was obtained from Kenya. Each virus was maintained separately in Mini Maize plants. The 14‐day‐old plants were inoculated with the viruses with a repeat inoculation 3 days later, the second and third youngest leaves collected 14 days after the first inoculation and processed for inoculum preparation. The virus inoculum was prepared by homogenising the leaves in a 10‐fold excess (w/v) of 0.1 mM phosphate buffer (pH 7.0) containing 0.1% (w/v) carborundum using a pestle and mortar. The homogenate was passed through a cheesecloth, debris discarded and filtrate used directly to inoculate the plants. The MLN inoculum was prepared by mixing MCMV and SCMV extracts in a 3:1 ratio (v/v). The MLN inoculum was rub‐inoculated on the 2nd and 3rd youngest leaves of the 14‐day‐old plants (V4 to V5 stage) using cotton swabs, with a repeat application three days later onto the same leaves. MLN symptoms were evaluated on a scale of 1 (no symptom, resistant or R) to 9 (plant death, susceptible or S) at 14, 16, 19, 21 and 24 days after the second inoculation.

#### ELISA assays

Starting at 20 days after MLN inoculation, four leaf discs (6 mm diameter) from the 2^nd^ youngest leaf with a visible bracket were sampled into megatiter tubes containing 3, 3‐mm glass beads and frozen in a − 80 °C freezer. The frozen tubes were shaken in a GenoGrinder at 1500 rpm for 30s and virus extracted in 0.6 mL, 1 mM phosphate buffer, pH 7.0. Following centrifugation at 4000 x g for 5 min, an aliquot of the supernatant was removed to measure protein concentration using the BCA method (BCA1, Sigma‐Aldrich). The extracts were generally diluted 120‐fold for SCMV and 2400‐fold for MCMV, respectively, for the ELISA assays. Double‐antibody sandwich ELISA assays were carried out for SCMV and MCMV following the manufacturer's protocol (140 475 and 140 775, BIOREBA, Switzerland). The relative virus concentration measured as absorbance above the control samples was normalised against the protein concentration.

#### RT‐PCR

Total RNA was extracted from the leaf tissue using the PureLink RNA Mini kit (12 183 025, Thermo Fisher) and first‐strand cDNA was synthesised using the SuperScript IV First‐Strand Synthesis System (18 091 050, Thermo Fisher) using 5 μg of total RNA in a 20 μL reaction volume. To amplify *eIF4E2* cDNA from CKL05022 and Mini Maize, a 5 μL first‐strand cDNA synthesis reaction was used in a 25 μL GoTaq PCR reaction (M3005, Promega). To amplify the *eIF4E2* cDNA from the *eif4e1‐KO/FL‐eIF4E2‐OX* (OX, overexpressing) Mini Maize plants where the OX gene was under the control of a *ZmUbi1* promoter, a 1 μL first‐strand cDNA synthesis reaction was used. Sanger sequencing was outsourced to Langebio Genomic Service Laboratory (Irapuato, Mexico). The same primers were used in all PCR reactions and for Sanger sequencing (F‐CCATGGCCGACGAGATTG, R‐GTCCCTTGTCCATCTTCTTTGC).

#### Protein structure prediction

The human eIF4E structure and its predicted binding to VPg and eIF4G were drawn based on known structures (Coutinho de Oliveira *et al*., 2019; Peter *et al*., 2015; Volpon *et al*., 2006). The human eIF4E was used as a template to predict the structures of the wildtype maize eIF4E2, mini maize eIF4E2 and the eIF4E2 variants that resulted from gene editing. The structures were predicted using the Swiss‐Model (https://swissmodel.expasy.org) and visualised using the UCSF Chimera software.

## Author contributions

ZW, FL and KSD conceptualised the study; ZW and FL collected the data and all three analyzed the data and wrote the manuscript. ZW and FL carried out gene editing, biochemical and molecular analyses and greenhouse screening. MJ, SH, LM, CH, EW and TJ provided molecular support, which included providing vectors for gene editing and sequencing of edited events. MC and IM assisted with plant growth, tissue transformation, regeneration and greenhouse screening. LMS and YB tested maize lines in the field in Kenya. KP, PB, and TJ were administrative points of contact. KSD conceived and supervised the project, wrote, revised and finalised the manuscript.

## Supporting information


**Figure S1.** Phylogenetic analysis of plant eukaryotic translation initiation/elongation factors. Geneious Prime software was used to build the phylogenetic tree of maize proteins along with known proteins from other plant species where their mutants had been shown to confer virus resistance ^1–22^. Two maize proteins, ZmeIF4E1 and ZmeIF4E2, were grouped with the known proteins for virus resistance from the largest number of plant species (green box). In addition to these two factors, we also selected eIF(iso)4E1 and eIF(iso)4E2 for further study (yellow box). We further selected four proteins in that grouped with the eIF4G proteins from other plant species (orange and purple boxes) but would be the subject of a separate study.
**Figure S2.** Editing of *eIF4E* genes in elite maize lines CKL05022 and CML536, and Mini Maize. The guide RNAs were designed to generate edits in the first exon. Nucleotide deletions are depicted with hashes (red), and additions are shown with respective bases under each sequence. Only the construct‐free events are listed. The events labelled ‘tested event’ were subjected to the MLN inoculation experiment as shown in Figure 2.
**Figure S3.** Editing of *eIF(iso)4E* genes in elite maize lines CKL05022 and CML536. See Figure S2 for details.
**Figure S4.** (a) Alignment of eIF4E1 and eIF4E2 proteins from Mini Maize, CML536 and CKL05022. The residues K194 and H204, which may facilitate the binding of the viral genome‐linked protein (VPg) to eIF4E are shown with green down‐arrows (see Figure S5 for details). (b) Alignment between human eIF4E and Mini Maize eIF4E1 and eIF4E2 proteins. The conserved residues involved in binding and stabilizing m7G‐cap as reported in Marcotrigiano *et al*. (1997) are shown with down arrows (red for tryptophan, blue for lysine/arginine, and orange for aspartate/glutamate). None of these residues occurs in the C‐terminal 38 amino acids, which likely explains the lack of effect of their deletion on translation of the host mRNA. In contrast, the lack of two key amino acids, lysine and histidine, in the Mini Maize eIF4E2 protein could explain the lack of binding of the VPg. It must also account for the lack of binding of 3′‐CITEs. The eIF4E1 and eIF4E2 proteins are 89% identical between CKL05022 and CML536. The eIF4E1 is shorter by two amino acids than the eIF4E2 protein (positions 20 and 21). In the C‐terminal 38‐aa (183–220) stretch (underlined in red), they differ only at two amino acids, both conservative substitutions (Ilu −183 to Val and Phe −192 to Leu).
**Figure S5.** Predicted structure of maize eIF4E2.The human eIF4E structure (a) and predicted VPg‐eIF4E‐eIF4G binding model (b) were drawn based on Coutinho de Oliveira *et al.*, 2019; Peter *et al.*, 2015; and Volpon *et al.*, 2006. The human eIF4E was used as template to predict the structures of the wildtype maize eIF4E2 (c), Mini Maize eIF4E2 (d), and one of the eIF4E2 variants from gene editing in an elite maize line (e). The structures were predicted using the Swiss‐Model (https://swissmodel.expasy.org) and visualized using the UCSF Chimera software. In the human eIF4E, residues K192 and H200 facilitate the interaction with the potato virus Y genome‐linked protein (PVY‐VPg) (gray). The corresponding residues 8 amino acids apart as in the human eIF4E are highlighted in the wildtype maize eIF4E2 (K196 and H204) but are absent from the Mini Maize protein because of the lack of the 4^th^ exon. Absence of the domain containing these key residues may destabilize the the interaction between the eIF4E and VPg. That would only explain the lack of replication of SCMV in Mini Maize and edited variants of CKL05022 and CML536, however. MCMV, a Tombusvirus, is not known to bind the eIF4E via VPg. It is believed instead to bind the eIF4E through 3′‐cap‐independent translation enhancers (3′‐CITEs), which consist of a variety of secondary structures resulting from the nucleotides in the 3′‐region of the viral genome (Simon and Miller, 2013). Regardless of the exact mechanism, the MCMV genome must not be able to recognize the modified eIF4E2 protein, just like in Mini Maize. Since the C‐terminal 38 amino acids of eIF4E are not required for eIF4G binding (eIF4G binds to the α‐helix 1 and 2 of eIF4E, purple), the translation initiation function of the eIF4E edited in the C‐terminal region is not affected for the host (maize) proteins.
**Figure S6.** RT‐PCR of *eIF4E2* from wildtype and *eif4e1‐KO/eIF4E2‐exon‐4ED* double edited events from CKL05022 and CML536 (a). (b) Sanger sequencing of the RT‐PCR products for CML536 from (a). For the events 1 and 2, it was a frameshift mutation. (c) In event 3, 8 bp were deleted from the genomic copy, including the base G of the AG intron/exon splicing motif (highlighted in green). The resulting mRNA was processed at an AG motif downstream of the disrupted splicing motif (highlighted in purple), resulting in an 18 bp (6 aa) in‐frame deletion. A closer examination of the RT‐PCR gel shows a corresponding, slight reduction in molecular mass of the amplified cDNA band of this event. Regardless of whether the deletions in the 4^th^ exon resulted in frameshift mutations (events 1 and 2) or in‐frame mutations (event 3) in the background of *eif4e1‐KO*, all the resulting events were all resistant to MLN (Figure 8).
**Table S1.** Single guide RNAs for different genes in the *eIF4E1* and *eIF4E2* genes for different maize lines.
[Correction added on 20 November 2024, after first online publication: the legends for supplementary figures S5 and S6 have been updated in this version.]

## Data Availability

All data supporting the findings of this study are available in the article and its Supplemental Information files. Plasmids used in this study can be provided under an applicable material transfer agreement to academic investigators for non‐commercial research.
