# Supplementary material for: Edited eukaryotic translation initiation factors confer resistance against maize lethal necrosis
Source: Plant Biotechnol J. 2024 Oct 15;22(12):3523–35. doi: 10.1111/pbi.14472 (PMC11606411; doi:10.1111/pbi.14472)
Supplement: Supplementary file 1 — Figure S1. Phylogenetic analysis of plant eukaryotic translation initiation/elongation factors. Geneious Prime software was used to build the phylogenetic tree of maize proteins along with known proteins from other plant species where their mutants had been shown to confer virus resistance 1–22. Two maize proteins, ZmeIF4E1 and ZmeIF4E2, were grouped with the known proteins for virus resistance from the largest number of plant species (green box). In addition to these two factors, we also selected eIF(iso)4E1 and eIF(iso)4E2 for further study (yellow box). We further selected four proteins in that grouped with the eIF4G proteins from other plant species (orange and purple boxes) but would be the subject of a separate study. Figure S2. Editing of eIF4E genes in elite maize lines CKL05022 and CML536, and Mini Maize. The guide RNAs were designed to generate edits in the first exon. Nucleotide deletions are depicted with hashes (red), and additions are shown with respective bases under each sequence. Only the construct‐free events are listed. The events labelled ‘tested event’ were subjected to the MLN inoculation experiment as shown in Figure 2. Figure S3. Editing of eIF(iso)4E genes in elite maize lines CKL05022 and CML536. See Figure S2 for details. Figure S4. (a) Alignment of eIF4E1 and eIF4E2 proteins from Mini Maize, CML536 and CKL05022. The residues K194 and H204, which may facilitate the binding of the viral genome‐linked protein (VPg) to eIF4E are shown with green down‐arrows (see Figure S5 for details). (b) Alignment between human eIF4E and Mini Maize eIF4E1 and eIF4E2 proteins. The conserved residues involved in binding and stabilizing m7G‐cap as reported in Marcotrigiano et al. (1997) are shown with down arrows (red for tryptophan, blue for lysine/arginine, and orange for aspartate/glutamate). None of these residues occurs in the C‐terminal 38 amino acids, which likely explains the lack of effect of their deletion on translation of the host mRNA. In contrast [file PBI-22-3523-s001.pdf]

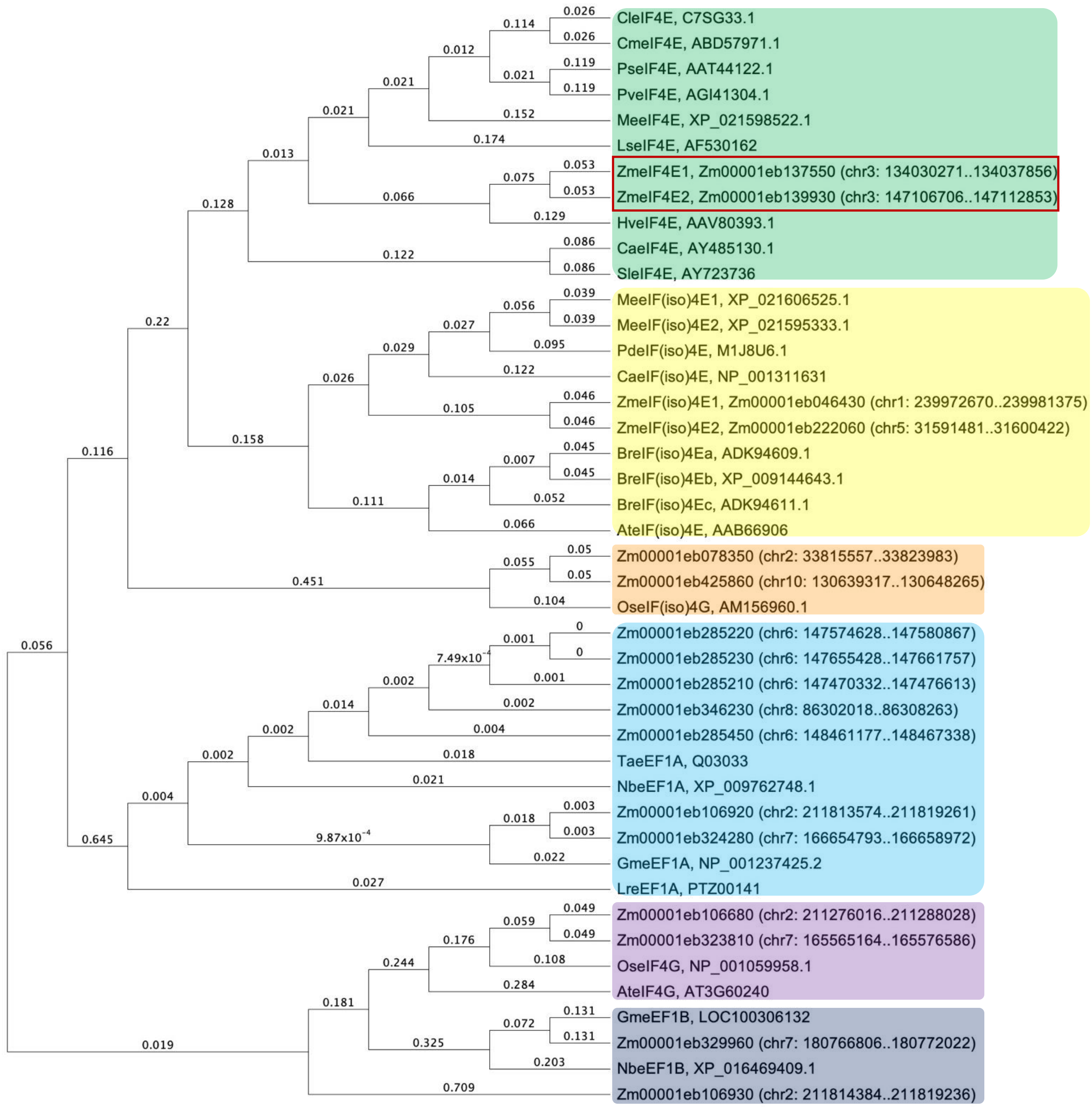

Figure S1. Phylogenetic analysis of plant eukaryotic translation initiation/elongation factors. Geneious Prime software was used to build the phylogenetic tree of maize proteins along with known proteins from other plant species where their mutants had been shown to confer virus resistance [1-22]. Two maize proteins, ZmeIF4E1 and ZmeIF4E2, grouped with the known proteins for virus resistance from the largest number of plant species (green box). In addition to these two factors, we also selected eIF(iso)4E1 and eIF(iso)4E2 for further study (yellow box). We further selected four proteins in that grouped with the eIF4G proteins from other plant species (orange and purple boxes) but would be the subject of a separate study.

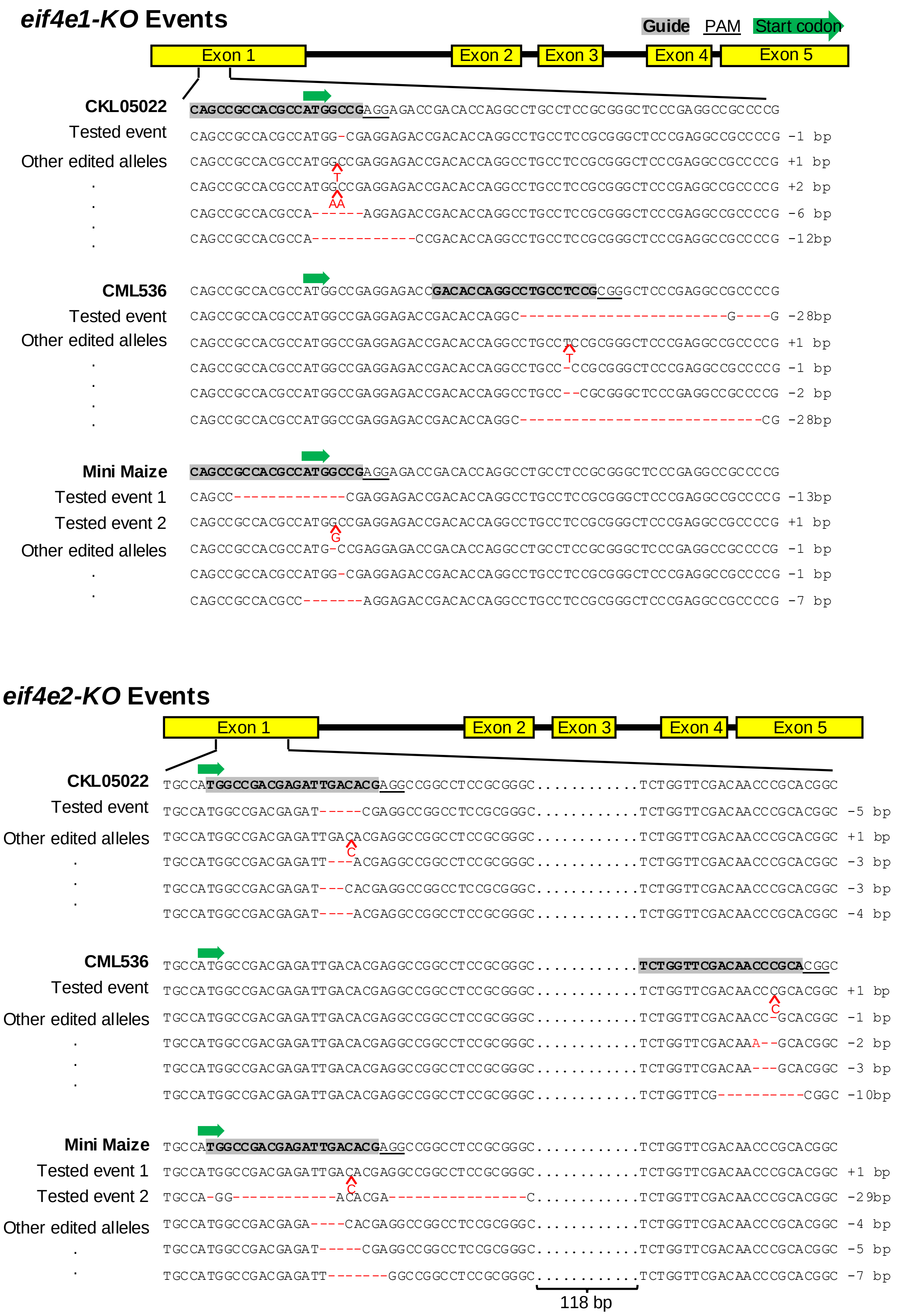

Figure S2. Editing of *eIF4E* genes in elite maize lines CKL05022 and CML536, and Mini Maize. The guide RNAs were designed to generate edits in the first exon. Nucleotide deletions are depicted with hashes (red) and additions are shown with respective bases under each sequence. Only the construct-free events are listed. The events labeled "tested event" were subjected to the MLN inoculation experiment as shown in Figure 2.

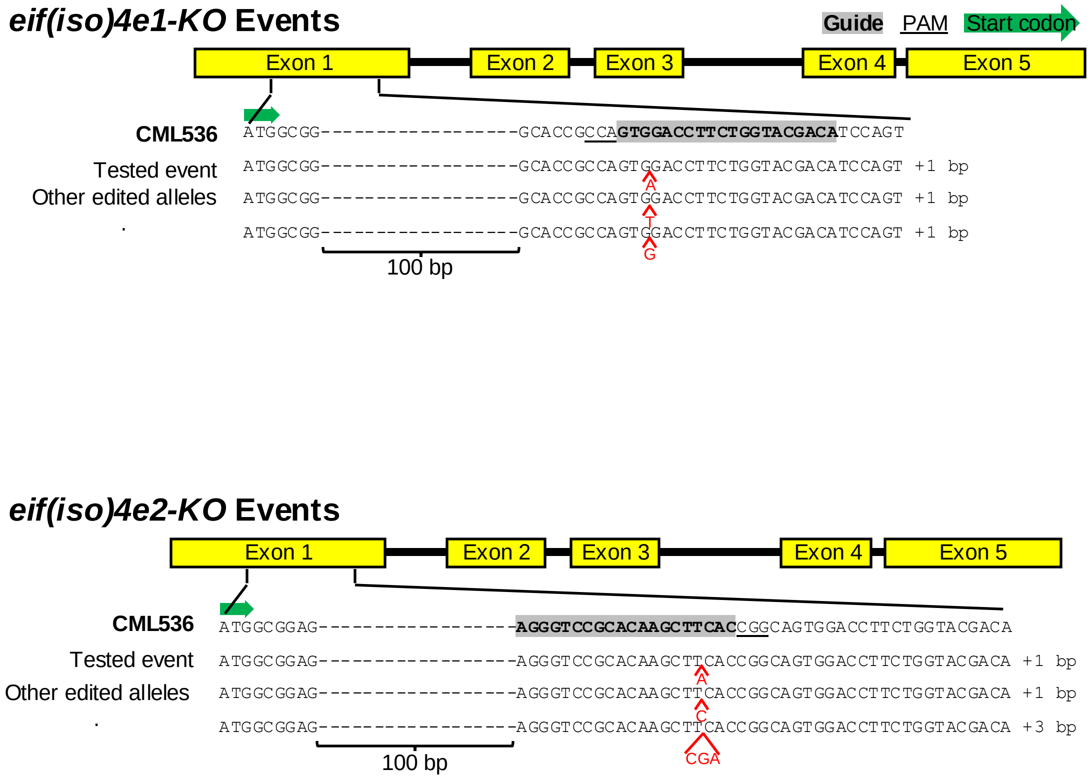

Figure S3. Editing of *eIF(iso)4E* genes in elite maize lines CKL05022 and CML536. See Figure S2 for details.

(a)

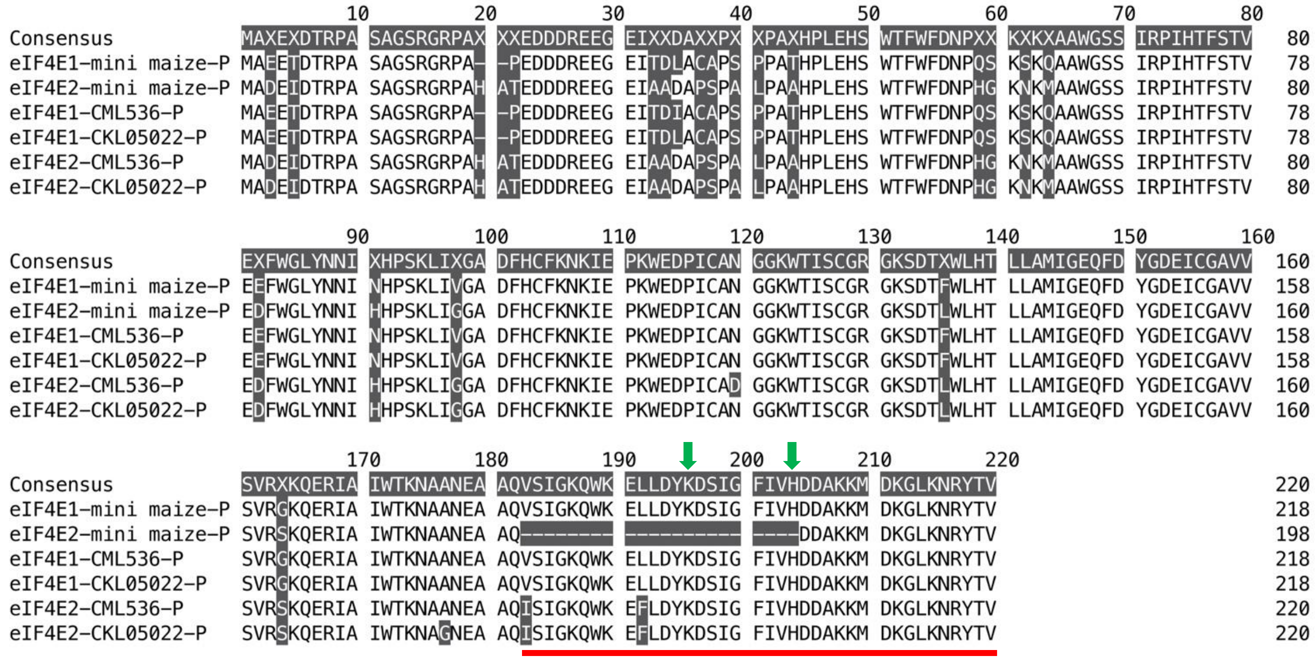

(b)

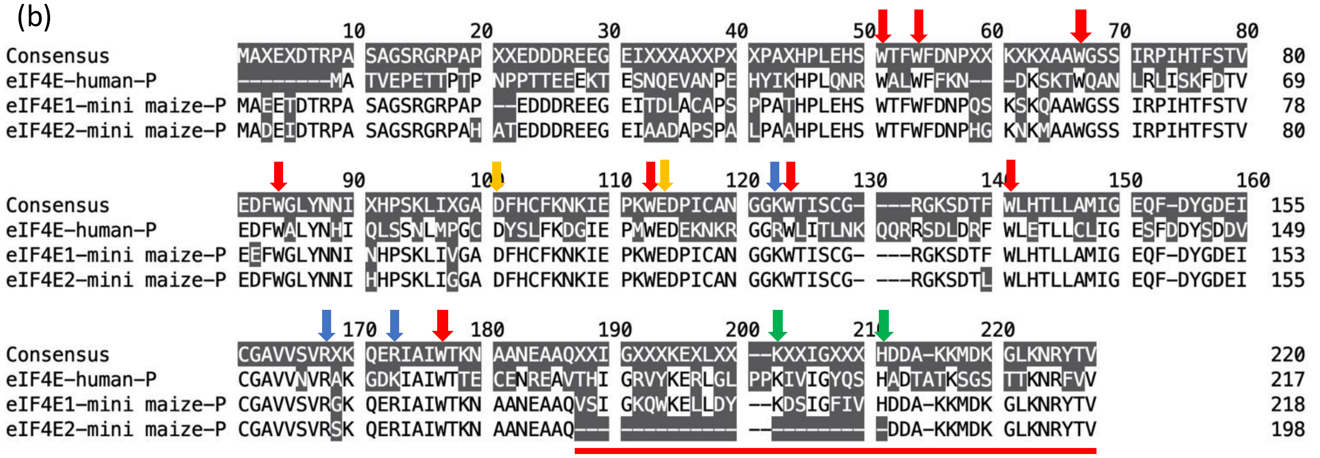

Figure S4. (a) Alignment of eIF4E1 and eIF4E2 proteins from Mini Maize, CML536 and CKL05022. The residues K194 and H204, which may facilitate the binding of the viral genome-linked protein (VPg) to eIF4E are shown with green down-arrows (see Figure S5 for details). (b) Alignment between human eIF4E and Mini Maize eIF4E1 and eIF4E2 proteins. The conserved residues involved in binding and stabilizing m7G-cap as reported in Marcotrigiano et al. (1997) are shown with down arrows (red for tryptophan, blue for lysine/arginine, and orange for aspartate/glutamate). None of these residues occurs in the C-terminal 38 amino acids, which likely explains the lack of effect of their deletion on translation of the host mRNA. In contrast, the lack of two key amino acids, lysine and histidine, in the Mini Maize eIF4E2 protein could explain the lack of binding of the VPg. It must also account for the lack of binding of 3'-CITEs.

The eIF4E1 and eIF4E2 proteins are 89% identical between CKL05022 and CML536. The eIF4E1 is shorter by two amino acids than the eIF4E2 protein (positions 20 and 21). In the C-terminal 38-aa (183-220) stretch (underlined in red), they differ only at two amino acids, both conservative substitutions (Ilu-183 to Val and Phe-192 to Leu).

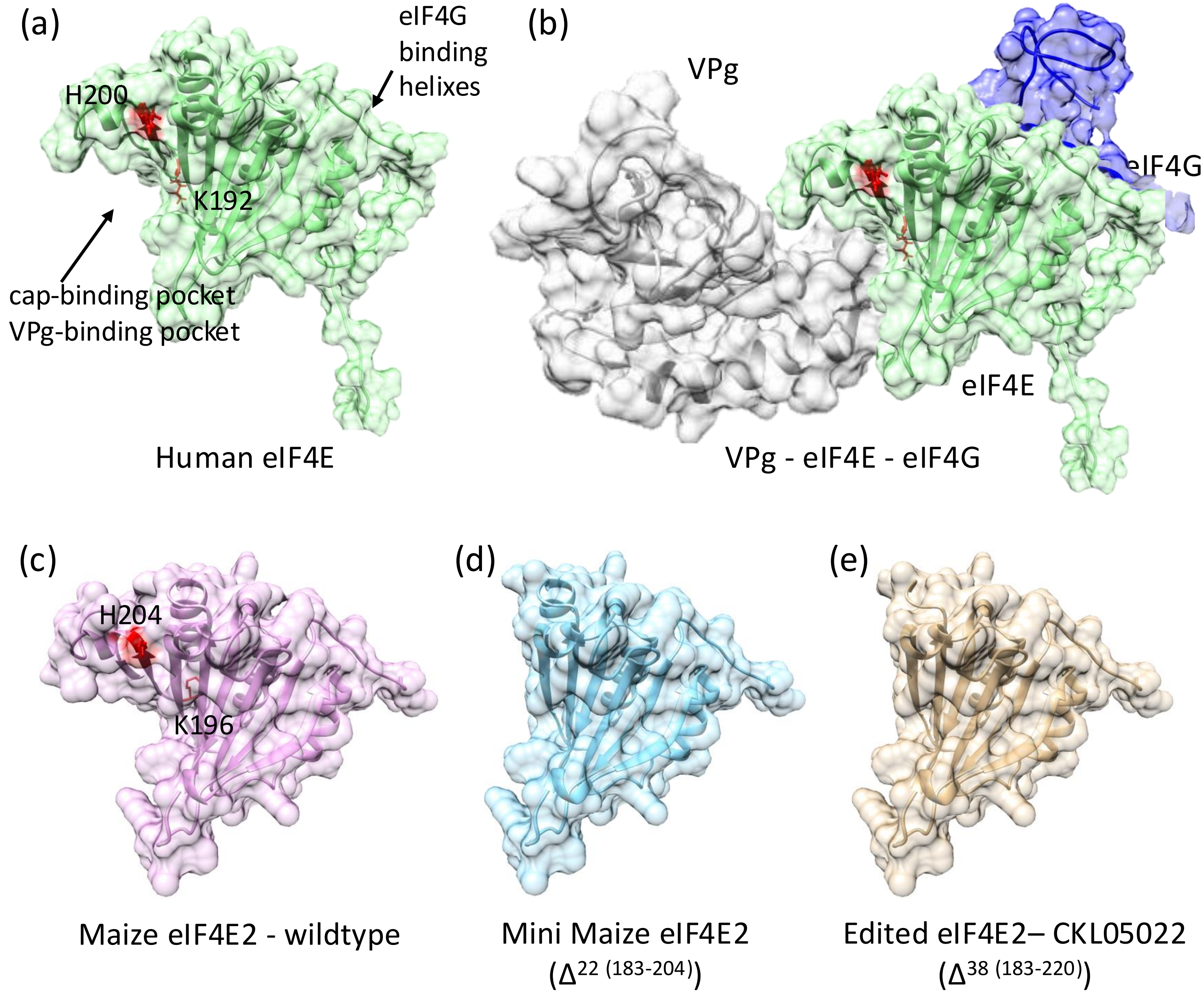

Figure S5. Predicted structure of maize eIF4E2.
The human eIF4E structure (a) and predicted VPg-eIF4E-eIF4G binding model (b) were drawn based on Coutinho de Oliveira et al., 2019; Peter et al., 2015; and Volpon et al., 2006 . The human eIF4E was used as template to predict the structures of the wildtype maize eIF4E2 (c), Mini Maize eIF4E2 (d), and one of the eIF4E2 variants from gene editing in an elite maize line (e). The structures were predicted using the Swiss-Model (https://swissmodel.expasy.org) and visualized using the UCSF Chimera software. In the human eIF4E, residues K192 and H200 facilitate the interaction with the potato virus Y genome-linked protein (PVY-VPg) (gray). The corresponding residues 8 amino acids apart as in the human eIF4E are highlighted in the wildtype maize eIF4E2 (K196 and H204) but are absent from the Mini Maize protein because of the lack of the 4th exon. Absence of the domain containing these key residues may destabilize the the interaction between the eIF4E and VPg. That would only explain the lack of replication of SCMV in Mini Maize and edited variants of CKL05022 and CML536, however. MCMV, a Tombusvirus, is not known to bind the eIF4E via VPg. It is believed instead to bind the eIF4E through 3'-cap-independent translation enhancers (3'-CITEs), which consist of a variety of secondary structures resulting from the nucleotides in the 3'-region of the viral genome (Simon and Miller, 2013). Regardless of the exact mechanism, the MCMV genome must not be able to recognize the modified eIF4E2 protein, just like in Mini Maize. Since the C-terminal 38 amino acids of eIF4E are not required for eIF4G binding (eIF4G binds to the α-helix 1 and 2 of eIF4E, purple), the translation initiation function of the eIF4E edited in the C-terminal region is not affected for the host (maize) proteins.

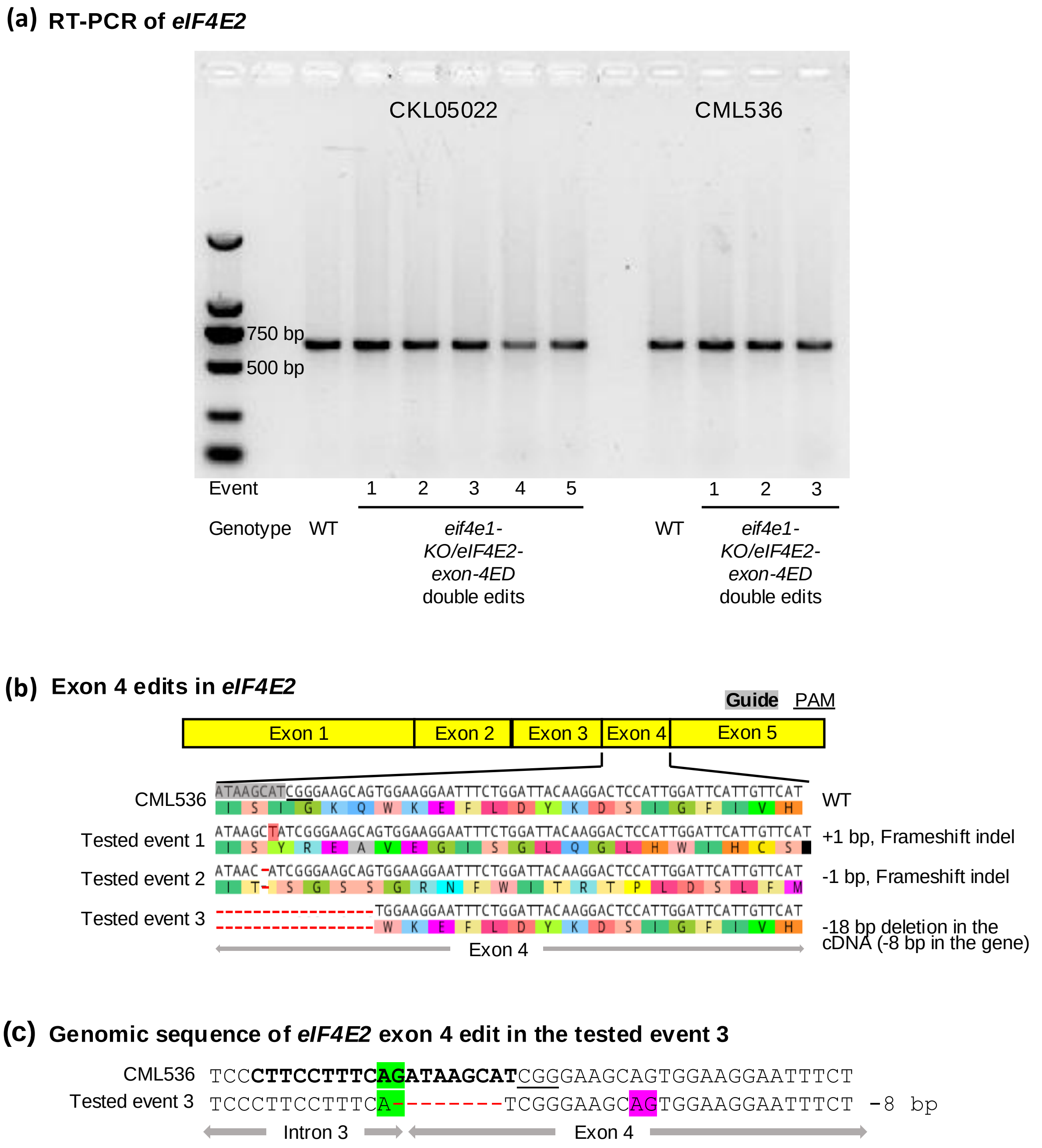

Figure S6. RT-PCR of *eIF4E2* from wildtype and *eif4e1-KO/eIF4E2-exon-4ED* double edited events from CKL05022 and CML536 (a). (b) Sanger sequencing of the RT-PCR products for CML536 from (a). For the events 1 and 2, it was a frameshift mutation. (c) In event 3, 8 bp were deleted from the genomic copy, including the base G of the AG intron/exon splicing motif (highlighted in green). The resulting mRNA was processed at an AG motif downstream of the disrupted splicing motif (highlighted in purple), resulting in an 18 bp (6 aa) in-frame deletion. A closer examination of the RT-PCR gel shows a corresponding, slight reduction in molecular mass of the amplified cDNA band of this event. Regardless of whether the deletions in the 4th exon resulted in frameshift mutations (events 1 and 2) or in-frame mutations (event 3) in the background of *eif4e1-KO*, all the resulting events were all resistant to MLN (Figure 8).

Table S1. Single guide RNAs for different genes in the *eIF4E1* and *eIF4E2* genes for different maize lines.

Target line	Target Gene	Guide sequence	Purpose
CKL05022 / Mini Maize	*eIF4E1*	AGCCGCCACGCCATGGCCG	Knockout
CKL05022 / Mini Maize	*eIF4E2*	GGCCGACGAGATTGACACG	Knockout
CML536	*eIF4E1*	GACACCAGGCCTGCCTCCG	Knockout
CML536	*eIF4E2*	TCTGGTTCGACAACCCGCA	Knockout
CKL05022 / CML536	*eIF4E2*	CTTCCTTTCAGATAAGCAT	Edit exon 4
